# MDHANet: Rethinking HOG as a Prior in Dense Networks with Self-Attention for Hyperspectral Image Classification

**DOI:** 10.3390/s26154731

**Published:** 2026-07-25

**Authors:** Hongwei Zhang, Yuanyuan Gui, Junjie Mou, Chao Lian

**Affiliations:** 1 School of Artificial Intelligence, China University of Mining and Technology-Beijing, Beijing 100083, China; 2310410332@student.cumtb.edu.cn (H.Z.); 2310410319@student.cumtb.edu.cn (J.M.); 2 State Key Laboratory of Space Information System and Integrated Application, Beijing 100095, China; 3 School of Information Science and Engineering, Northeastern University, Shenyang 110819, China

**Keywords:** hyperspectral image classification, histogram of oriented gradients (HOG), self-attention, dense connections, multiscale feature extraction, spatial structure prior

## Abstract

Convolutional neural network (CNN)-based methods have been widely adopted in hyperspectral image (HSI) classification tasks and have demonstrated superior performance due to their potent nonlinear fitting capabilities. However, as network depth increases, existing methods suffer from significant issues such as gradient vanishing and information loss, which limit further improvements in model performance. Meanwhile, current approaches primarily design entirely new network architectures to extract abstract features but fail to fully exploit the explicit gradient prior information inherent in the data, which affects model performance in complex scenarios. To address these issues, this paper proposes a Multiscale Dense HOG-driven Self-Attention Network (MDHANet) that incorporates the Histogram of Oriented Gradients (HOG) as prior knowledge. First, the model employs three densely connected branches at different scales to obtain diverse multi-level feature representations through multi-scale feature reuse. Furthermore, since HOG can explicitly encode the distribution of gradient orientations and magnitudes in local neighborhoods and accurately characterize the feature distribution, a dynamic HOG-driven self-attention module is designed. Within this module, a HOG prior-based feature rearrangement strategy together with a dual-branch self-attention mechanism enables the network to more effectively learn the spatial information of the data, enhancing the extraction of discriminative spatial–structure features and further improving classification performance. Extensive experimental results on multiple datasets demonstrate that the proposed method outperforms existing state-of-the-art approaches. Extensive experimental results on four HSI datasets (IP, LK, HH, and HC) demonstrate that the proposed method outperforms existing state-of-the-art approaches, achieving OA of 99.13%, 97.32%, 99.02%, and 99.24%, respectively, with improvements of 0.83%, 0.41%, 2.69%, and 2.47% over the best competing methods.

## 1. Introduction

Hyperspectral images (HSIs) simultaneously contain information from hundreds of contiguous spectral bands and spatial distribution data, which can accurately reflect subtle differences in land features that are difficult to distinguish using traditional multispectral imagery. Consequently, they have attracted substantial research interest [1,2,3,4]. As a key task in the field of hyperspectral image analysis, hyperspectral image classification (HSIC) aims to assign a unique land cover class label to each pixel in an image [5]. This is a prerequisite for many practical applications, such as agricultural monitoring, urban planning and management, mineral mapping, and environmental protection [6,7,8,9].

Over the past few decades, scholars have conducted extensive research on the HSIC task, yielding a series of classification frameworks based on traditional machine learning approaches such as support vector machine (SVM) [10,11,12], random forest (RF) [13,14], multivariate logistic regression [15,16], K-nearest neighbor (KNN) [17], and sparse representation-based classifier (SRC) [18,19,20]. However, the features extracted by these methods rely on manually designed rules and parameters. This makes it difficult to effectively capture the deep abstract semantics contained in data, which leads to a bottleneck in classification performance.

In recent years, deep learning has provided new perspectives and tools for addressing these challenges. Unlike traditional shallow models, deep neural networks possess powerful hierarchical feature learning and nonlinear fitting capabilities, enabling them to automatically abstract and extract deep semantic features layer-by-layer from raw high-dimensional data [21,22]. Among the various architectures, convolutional neural networks (CNNs) have been extensively studied. Representative CNN architectures for HSIC include 2D CNNs that operate on spatial patches, 3D CNNs that process spatial-spectral cubes jointly, and hybrid architectures that combine both 2D and 3D convolutions to reduce the high computational cost of pure 3D convolutions.

While CNN-based methods have achieved some success in local feature extraction, the limited receptive fields of convolutional kernels make it difficult for models to effectively capture long-range dependencies between distant pixels in hyperspectral images [23,24]. This global contextual information is crucial for accurately identifying land cover classes that share similar features but are spatially dispersed. Self-attention mechanisms directly model global dependencies by computing the similarity between any two elements in the input sequence and generating a global dependency matrix, enabling the output features at each position to directly aggregate information from all other positions [25]. Accordingly, self-attention has been introduced into HSIC as a way to break through the aforementioned bottleneck.

Although methods based on CNNs and self-attention mechanisms have achieved success, existing research still faces two critical challenges. First, models are becoming increasingly complex in order to achieve stronger feature representation. As network depth increases, simply stacked CNNs are highly prone to gradient vanishing, making deep networks more difficult to train effectively and potentially causing performance degradation [26]. Although methods such as residual connections can mitigate gradient vanishing to some extent [26], their skip connections are relatively simple, merely transferring features between adjacent or specific layers and failing to fully reuse the rich hierarchical features across different layers [27]. Second, the vast majority of existing deep learning classification frameworks concentrate on enabling models to automatically learn complex mappings from inputs to outputs through meticulously designed network modules. However, this paradigm overlooks the considerable potential value of explicit low-level prior knowledge inherent in hyperspectral data. This knowledge can be used to guide model learning, and ignoring it can introduce a limit on performance improvements in complex scenarios.

To overcome the above limitations, we propose a Multiscale Dense HOG-driven Self-Attention Network (MDHANet) that incorporates Histogram of Oriented Gradients (HOG) as prior knowledge. First, we adopt a feature extraction backbone with three branches at different scales. Dense connections are introduced between convolutional layers within each branch to ensure the efficient flow of shallow details and deep semantic information throughout the network. This mitigates the vanishing gradient problem, maximizes feature reuse, and provides a richer feature foundation for the subsequent self-attention modules. Second, the spatial structural patterns of different ground objects serve as critical visual cues for distinguishing their categories; for instance, farmlands typically exhibit regular parcel boundaries, airport runways or main roads present narrow linear extensions, and most buildings display distinct orthogonal edges. HOG can explicitly and efficiently encode the orientation and magnitude distribution of gradients within local neighborhoods, enabling precise perception of spatial structure and edge direction in images [28]. Based on this, we design a dynamic HOG-driven attention module to enhance the discriminative power of spatial–structure features. Rather than simply incorporating static, manually computed HOG features, this module reformulates them into a learnable and differentiable component. Specifically, the HOG descriptor serves as a spatial structure prior, driving the model to dynamically reorganize features within local windows for efficient convolution. Subsequent global ordering then aggregates pixels that are spatially dispersed but share similar gradient patterns along the feature dimension. Finally, the model fuses these components through two parallel self-attention branches employing different strategies, producing an enhanced feature representation that captures both local details and global structural information.

The main contributions of this article are summarized as follows:

1. We propose MDHANet, an end-to-end hyperspectral image classification network that integrates multi-scale dense connections with explicit gradient priors. Leveraging the efficient reuse of multi-level features, this method innovatively incorporates HOG into the self-attention module as a spatial structure prior, effectively addressing the shortcomings of purely data-driven models in perceiving and utilizing the spatial structural information of ground objects.
2. We construct a multi-scale densely connected feature extraction network. This network incorporates a dense connection mechanism within its parallel branches, providing subsequent modules with richer and more complementary information while effectively mitigating the vanishing gradient problem in deep networks.
3. We design a novel DHOGSA module that uses HOG descriptors of the data as explicit priors to guide the module’s operation. The local feature rearrangement process enables the convolution kernels to capture homogeneous structures in a single pass while preserving spatial consistency. The global sorting strategy directs the model to focus on pixels with similar characteristics. The dual-branch self-attention mechanism further captures and fuses local details and long-range dependencies. These designs provide the model with strong perception and discrimination capabilities for the spatial structure information of ground objects.

The rest of this article is organized as follows: Section 2 reviews related work; Section 3 describes the overall framework and detailed implementation of the proposed method; Section 4 presents extensive experiments and analysis; finally, Section 5 summarizes the work and concludes this article.

## 2. Related Work

### 2.1. CNN-Based Methods for HSIC

Makantasis et al. [22] were one of the earliest to introduce the 2D-CNN paradigm into hyperspectral classification, confirming the importance of spatial neighborhood information for improving classification accuracy. In this approach, spatial neighborhood blocks are used as input to capture local contextual information. Liu et al. [29] further combined 2D-CNNs with null spectrum superpixel segmentation and kernel extreme learning machine to achieve efficient classification on downscaled spectral and spatial features. Although 2D-CNNs can effectively extract spatial information, they struggle to fully exploit the continuous dependencies among spectral bands.

To simultaneously capture both the spatial and spectral features of hyperspectral data, researchers have incorporated 3D-CNNs into HSIC. Three-dimensional convolutional kernels slide along both spatial and spectral dimensions, naturally preserving spectral continuity and capturing richer joint features. The LGDRNet proposed by Chen et al. [30] further advances this direction by introducing a dynamic 3D convolutional module that generates differentiated convolutional weights based on different input samples, enhancing the network’s attention to key pixels in 3D samples. Beyond standard 3D convolutions, the incorporation of dense connectivity has also been explored to facilitate feature reuse. The WCNet proposed by Li and Ye [31] utilizes a 3D DenseNet as its backbone to extract joint spatial–spectral features. By introducing a 3D wavelet transform, it expands the effective receptive field of the convolutional kernels, enabling the network to flexibly focus on multi-scale spatial structures. However, this multi-scale capability is realized through a single-branch serial architecture, where features of different scales are processed sequentially within a single network flow. In contrast, a parallel multi-branch design can simultaneously extract features at different scales at the same network depth, providing richer and more diverse feature representations for subsequent modules.

Notably, hybrid architectures combining 2D and 3D convolutions have been proposed to achieve powerful feature extraction while reducing the high computational cost of pure 3D convolutions. Depending on how 2D and 3D convolutions are organized, these architectures can be broadly classified into serial and parallel designs. In the serial designs, Roy et al. [32] proposed a hybrid convolutional network (HybridSN) that places 3D convolutions in the shallow layers to extract low-level joint spectral–spatial features while switching to 2D convolutions in the deep layers to further extract high-level spatial semantics. This approach effectively reduces the number of parameters and computational overhead in the deep network while maintaining classification accuracy. Regarding parallel designs, Chen et al. [33] proposed a 2D–3D parallel convolutional network that employs a dual-branch parallel architecture: one branch enhances target spectral features using a hyperspectral target detection algorithm, followed by 2D-CNN learning of spatial features, while the other branch employs a 3D CNN to extract joint features after PCA dimensionality reduction. The fusion of these two feature pathways achieves complementary advantages of spectral enhancement and joint spatial–spectral modeling.

Despite the significant progress made by CNN-based methods in local feature extraction, the limited receptive fields of convolutional kernels restrict their ability to capture long-range dependencies between distant pixels, and the vanishing gradient problem further limits the performance of deeper models as network depth increases.

### 2.2. Self-Attention-Based Methods for HSIC

In recent years, self-attention-based methods have been widely explored for hyperspectral image classification. For example, in the spectral dimension, the SpectralFormer proposed by Hong et al. [34] takes the spectral sequences of hyperspectral pixels as input, captures local spectral information through grouped spectral embeddings, and utilizes the self-attention layers in the transformer to model long-range dependencies between spectral bands. In the spatial dimension, the PSFormer proposed by Zou et al. [35] constructs a transformer backbone network with superpixels as nodes. By establishing global spatial correlations between superpixels through the self-attention mechanism, it overcomes the limitation of traditional methods which can only propagate information between adjacent superpixels. In recent years, researchers have attempted to combine self-attention with CNN architectures to balance local details and global context. For example, Hu et al. [36] introduced a transformer-based self-attention module at the end of a multi-path parallel 3D-CNN and designed a cross-attention fusion mechanism to enable bidirectional interaction between the local spectral–spatial features extracted by the CNN and the global dependencies modeled by the self-attention. Zhong et al. [37] proposed LCTNet, in which the self-attention mechanism of a dynamic sparse transformer module captures global dependencies from shallow features extracted by CNNs. By utilizing an entropy-guided gating strategy to adaptively adjust attention sparsity, the method enhances classification accuracy while maintaining a lightweight architecture. Furthermore, the RAT-MPC proposed by Sun et al. [38] adopts a parallel dual-branch framework. The self-attention mechanism in the Agent Re-Attention Transformer (RAT) branch models global spectral dependencies, while the Multi-Scale Partial Convolution (MPC) branch extracts local spatial features. The fusion of these two feature streams effectively balances the representation of local details and global context.

Despite the advantages of self-attention-based methods in global modeling, their computational complexity grows quadratically with spatial resolution, limiting their application on large-scale hyperspectral data.

### 2.3. Explicit Priors in Deep Learning Architectures

In the development of deep learning, the incorporation of prior knowledge has proven effective in constraining model training, improving generalization, and enhancing the recognition of key structural features. In the field of medical image processing, Dong and Basu [39] utilized gradient-based XAI to extract feature importance distributions from clean medical images as explicit priors, converting them into constraint signals via a feature-preserving loss function to guide the denoising network in preserving critical diagnostic features while removing noise. Banik [40] introduced spatial and geometric priors into hyperspectral unmixing tasks, achieving superior abundance estimation accuracy on real-world datasets by imposing minimum simplex volume constraints on the network and leveraging the implicit priors inherent in the network architecture itself.

In HSI analysis tasks, HOG, as a classic gradient feature descriptor, has been widely used to extract spatial structural information. Xie [41] applied it to hyperspectral face recognition by computing gradient direction histograms for each band image to construct feature vectors, combined with a collaborative representation classifier (CRC) for classification. Tang et al. [42] extended HOG to three-dimensional space, extracting gradient histograms on three orthogonal planes (XY, XZ, YZ) and fusing them via fuzzy integration into a 3D-FHOG descriptor, which was then concatenated with deep features for classification. Zhang et al. [43] proposed adaptive-scale 3D HOG features that simultaneously compute gradient direction histograms in spatial and spectral dimensions, adapting to target size variations through a multi-scale strategy and finally fusing the response maps with deep feature response maps via weighted averaging for hyperspectral video target tracking. Although the above methods attempt to incorporate HOG features into networks, their core approach remains the design of more refined handcrafted descriptors. HOG consistently exists as a fixed and manually computed feature, and its interaction with deep networks is limited to feature input, concatenation, or response map fusion.

One underexplored avenue consists of transforming HOG from a fixed handcrafted descriptor into a differentiable learnable prior that can be embedded into the network architecture to guide the model’s learning and decision-making process and achieve end-to-end joint optimization. This paper rethinks HOG as a learnable spatial structure prior and embeds it into a self-attention mechanism. This enables the network to adaptively learn how to utilize gradient information for spatial structure perception, achieving end-to-end hyperspectral image classification.

## 3. Method

### 3.1. Overall Framework

**Figure 1:**
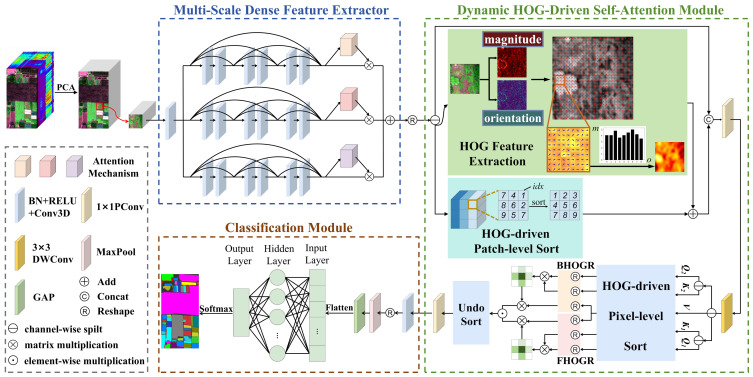
Overview of proposed MDHANet architecture.

The overall architecture of the proposed MDHANet is illustrated in Figure 1, which consists of three core modules: a Multi-scale Dense Feature Extractor (MDFE), a Dynamic HOG-driven Self-Attention module (DHOGSA), and a classification module. First, after reducing the dimensionality of the raw hyperspectral data via PCA, a 3D neighborhood cube $I\in R^{S\times S\times B}$ centered on the target pixel is extracted as the network input, where S×S denotes the spatial neighborhood size and *B* represents the number of retained spectral bands. Subsequently, the MDFE extracts features under varying receptive fields through three parallel branches, which are then fused to yield a multi-granularity feature representation. Next, the DHOGSA module utilizes HOG as an explicit spatial structural prior to further enhance the discriminative power of features for spatial structures of ground objects through a two-stage feature rearrangement strategy and a dual-branch self-attention mechanism. Finally, the prediction probability for each category is generated via a fully connected layer and a softmax function.

### 3.2. Multi-Scale Dense Feature Extractor

Land cover objects in HSI exhibit substantial variations in spatial scale, making it difficult for a single fixed receptive field to effectively capture features across such diverse scales. Moreover, HSI contains hundreds of spectral bands, and accurate classification typically requires sufficiently deep networks to model the complex spectral–spatial relationships inherent in the data. However, as network depth increases, gradient vanishing and information loss become critical obstacles that limit model performance. To address these issues, we construct an MDFE that combines a multi-branch parallel design with dense connections.

The MDFE consists of an initial 3D convolutional layer, three parallel branches, and attention units at the end of each branch. The input first passes through an initial 3D convolutional layer, which includes batch normalization, ReLU activation, and 3×3×3 convolutional kernels, resulting in extracted shallow spectral–spatial features X:

(1) $$X=\mathrm{ReLU}\left(\mathrm{BN}\left({\mathrm{Conv}}_{3\times 3\times 3}\left(I\right)\right)\right)$$

where $X\in R^{C\times D\times H\times W}$, *C* denotes the number of channels, and *D* and H×W are the spectral dimension and spatial size after the initial convolution, respectively. Then, X is fed into three parallel branches, each using kernel sizes 3, 5, and 7. Taking the *i*-th branch as an example, its detailed structure is described below.

**(1) Dense Connection Unit (DCU).** This unit consists of three layers, each of which adopts a cascaded structure of spatial and spectral 3D convolutions to reduce the model parameters. With X as the unit input, the first layer generates a new feature $Y_{i}^{\left(1\right)}$ through spatial and spectral convolutions:

(2) $$Y_{i}^{\left(1\right)}=\mathrm{ReLU}\left(\mathrm{BN}\left({\mathrm{Conv}}_{n\times 1\times 1}\left(\mathrm{ReLU}\left(\mathrm{BN}\left({\mathrm{Conv}}_{1\times n\times n}\left(X\right)\right)\right)\right)\right)\right)$$

where $Y_{i}^{\left(1\right)}\in R^{32\times D\times H\times W}$, since the output channels of both the spatial and spectral convolutions are set to 32.

The second layer takes the concatenation of $Y_{i}^{\left(1\right)}$ and the original input X along the channel dimension as its input, yielding $Y_{i}^{\left(2\right)}$ via an identical structure:

(3) $$Y_{i}^{\left(2\right)}=\mathrm{ReLU}\left(\mathrm{BN}\left({\mathrm{Conv}}_{n\times 1\times 1}\left(\mathrm{ReLU}\left(\mathrm{BN}\left({\mathrm{Conv}}_{1\times n\times n}\left(\left[Y_{i}^{\left(1\right)},X\right]\right)\right)\right)\right)\right)\right)$$

where [·] denotes the concatenation operation along the channel dimension.

The third layer utilizes the concatenation of $Y_{i}^{\left(1\right)}$, $Y_{i}^{\left(2\right)}$, and the original input X as its input to obtain $Y_{i}^{\left(3\right)}$:

(4) $$Y_{i}^{\left(3\right)}=\mathrm{ReLU}\left(\mathrm{BN}\left({\mathrm{Conv}}_{n\times 1\times 1}\left(\mathrm{ReLU}\left(\mathrm{BN}\left({\mathrm{Conv}}_{1\times n\times n}\left(\left[Y_{i}^{\left(1\right)},Y_{i}^{\left(2\right)},X\right]\right)\right)\right)\right)\right)\right).$$

The final output $U_{i}$ of the DCU is formed by concatenating the outputs of all individual layers with the original input along the channel dimension:

(5) $$U_{i}=\left[Y_{i}^{\left(1\right)},Y_{i}^{\left(2\right)},Y_{i}^{\left(3\right)},X\right]$$

where $U_{i}\in R^{C_{dc}\times D\times H\times W}$ and $C_{dc}=160$.

**(2) Attention Unit.** The above output $U_{i}$ then enters a three-branch attention unit, which generates adaptive weights from the spectral, spatial, and channel dimensions to suppress noise and redundancy.

Spectral attention compresses all channel and spatial information of each spectral band into a single scalar via global average pooling (GAP), followed by spectral convolution and Sigmoid normalization to produce the spectral attention weight $W_{se}$:

(6) $$W_{se}=\sigma \left({\mathrm{Conv}}_{n\times 1\times 1}\left(\mathrm{Permute}\left(\mathrm{GAP}\left(\mathrm{Permute}(U_{i})\right)\right)\right)\right)$$

where Permute(·) swaps the spectral and channel dimensions, σ(·) is the Sigmoid function, and $W_{\mathrm{se}}\in R^{1\times D\times 1\times 1}$.

Spatial attention applies average pooling along the spectral dimension, followed by a spatial convolution and Sigmoid normalization to produce the weight $W_{sa}$:

(7) $$W_{sa}=\sigma \left({\mathrm{Conv}}_{1\times n\times n}\left(\mathrm{AvgPool}(U_{i})\right)\right).$$

Channel attention performs GAP over the spectral and spatial dimensions, flattens the result into a 2D vector, models global dependencies among channels via two fully connected layers, and finally normalizes the output to obtain $W_{ca}$:

(8) $$W_{ca}=\sigma \left(\mathrm{Reshape}\left(W_{2}\cdot \left(W_{1}\cdot \mathrm{Flatten}\left(\mathrm{GAP}(U_{i})\right)\right)\right)\right)$$

where $W_{1}\in R^{160\times (160/r)}$, $W_{2}\in R^{(160/r)\times 160}$ are learnable weights of the fully connected layers and *r* is the reduction ratio.

$W_{se}$, $W_{sa}$ and $W_{ca}$ are fused into a comprehensive weight tensor W via element-wise multiplication under a broadcasting mechanism:

(9) $$W=W_{se}⊙W_{sa}⊙W_{ca}.$$

The attention-enhanced output $F_{i}$ of the *i*-th branch is obtained by multiplying the original feature with this weight tensor:

(10) $$F_{i}=U_{i}⊙W.$$

**(3) Multi-Scale Feature Fusion.** The outputs $F_{1}$, $F_{2}$, and $F_{3}$ of the three branches are fused via element-wise addition to yield the final module output $F_{msd}$:

(11) $$F_{msd}=F_{1}⊕F_{2}⊕F_{3}$$

where $F_{msd}\in R^{C_{msd}\times D\times H\times W}$, $C_{msd}=C_{dc}$.

### 3.3. Dynamic HOG-Driven Self-Attention Module

Although MDFE captures rich hierarchical features, the practical characteristics of HSIs still bring challenges to subsequent processing. On one hand, HSIs may suffer from interference caused by illumination and local shadows during acquisition. On the other hand, the spatial neighborhoods in HSIs are usually mixed with a large number of irrelevant pixels. Because HOG exhibits extreme robustness against local illumination variations and since we aim to design an operation that aggregates spatially dispersed pixels in the feature dimension, we propose a dynamic HOG-driven self-attention (DHOGSA) module that integrates HOG into the self-attention calculation as an explicit spatial structural prior. The DHOGSA module consists of three key components: local dynamic range convolution (LDRConv), HOG-driven feature sorting, and dual-branch self-attention.

First, input preprocessing is performed in which $F_{msd}$ is reshaped into a three-dimensional feature map G:

(12) $$G={\mathrm{Reshape}}_{\left(C_{msd}\cdot D,H,W\right)}\left(F_{msd}\right)$$

where $G\in R^{C_{all}\times H\times W}$ and $C_{all}=C_{msd}\times D$.

**(1) Local Dynamic Range Convolution.** The static receptive field of standard convolution struggles to focus on pixel groups with similar structural characteristics, often introducing large amounts of irrelevant information. To address this, we design LDRConv, which dynamically reorganizes the pixel order within a local window according to the gradient distribution of the input features, enabling the convolution kernel to efficiently operate on sets of pixels with consistent structures.

**Figure 2:**
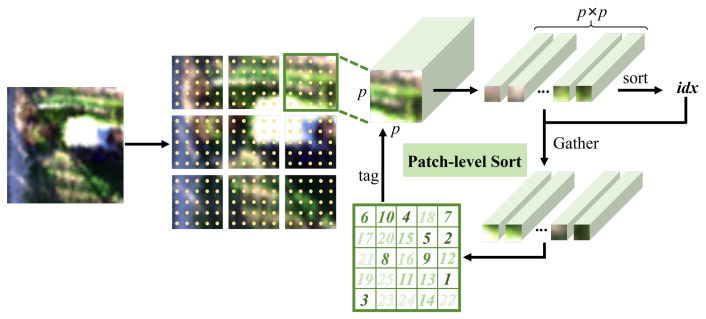
Patch-level sorting. Pixel indices within a window are constrained to contiguous values within that local neighborhood, as the sorting is confined to each individual patch.

Specifically, G is evenly split along the channel dimension into $G_{1}$ and $G_{2}$. HOG-driven block-level feature rearrangement is performed on $G_{1}$, while $G_{2}$ remains unchanged to preserve the original representation capability. For $G_{1}\in R^{C_{all}/2\times H\times W}$, the Sobel operator computes the gradient magnitude *m* and orientation *o* for each channel:

(13) $$g_{x}={\mathrm{Sobel}}_{x}\left(G_{1}\right),\;g_{y}={\mathrm{Sobel}}_{y}\left(G_{1}\right)$$

(14) $$m=\sqrt{g_{x}^{2}+g_{y}^{2}},\;o=\left\lfloor \frac{\mathrm{atan}2(g_{y},g_{x})+\pi }{2\pi }\times N_{bin}\right\rfloor$$

where $N_{bin}$ is the number of HOG orientation bins (set to 9). Then, $G_{1}$ is divided into p×p local patches. Within each patch, each pixel is assigned a sorting score computed as follows:

(15) $$S_{patch}\left(x,y\right)=m\left(x,y\right)\cdot \left(o\left(x,y\right)+1\right)$$

where m(x,y) and o(x,y) are the gradient magnitude and orientation index at pixel (x,y), respectively. The rationale behind this scoring formulation is to couple gradient magnitude and orientation into a unified scalar metric for local structural similarity. Within each local patch, pixels with similar edge orientations and intensities are assigned numerically close scores. This allows the subsequent patch-level sorting operation to effectively cluster these structurally homogeneous pixels together without disrupting critical local semantic and geometric information. The scoring allows the convolution kernels to perceive these consistent pixels in a single pass rather than dispersing their limited parameters across other heterogeneous pixels, significantly improving the purity of local spatial feature extraction. The overall process is illustrated in Figure 2.

Meanwhile, HOG features are computed within each local block; after being projected via a 1×1 convolution and bilinear interpolated, a spatially enhanced map is generated and incorporated:

(16) $${\tilde{G}}_{1}={\mathrm{Sort}}_{patch}\left(G_{1}\right)+{\mathrm{HOG}}_{\theta }\left(G_{1}\right)$$

where the patch-level pixel rearrangement operation based on the above scores $S_{\mathrm{patch}}$ is denoted as ${\mathrm{Sort}}_{\mathrm{patch}}\left(\cdot \right)$ and ${\mathrm{HOG}}_{\theta }\left(\cdot \right)$ represents the learnable HOG feature projection operation.

Finally, ${\tilde{G}}_{1}$ and $G_{2}$ are concatenated along the channel dimension and cross-channel information is fused through a 1×1 pointwise convolution and a 3×3 depthwise convolution, yielding the final output $G_{LDR}\in R^{C_{LDR}\times H^{\prime }\times W^{\prime }}$ of LDRConv:

(17) $$G_{LDR}={\mathrm{DWConv}}_{3\times 3}\left({\mathrm{PConv}}_{1\times 1}\left([{\tilde{G}}_{1},G_{2}]\right)\right).$$

**(2) HOG-Driven Feature Sorting.** The previous LDRConv performs pixel rearrangement within local patches to enhance intra-patch structural consistency. On this basis, the present module further performs global sorting to align pixels with similar structures at the global level. The process is described below.

The previous convolution operation mapped the concatenated features into the following five groups of feature maps:

(18) $$Q_{1},K_{1},Q_{2},K_{2},V={\mathrm{Chunk}}_{5}\left(G_{LDR}\right)$$

where ${\mathrm{Chunk}}_{5}\left(\cdot \right)$ denotes even splitting of the feature map into five groups along the channel dimension; $Q_{i}$, $K_{i}$, V are the query, key, and value matrices, respectively, with shape $R^{C^{\prime }\times H^{\prime }\times W^{\prime }}$, where $C^{\prime }=C_{LDR}/5$.

Then, global sorting indices are generated from the information-rich value features V to globally reorder the feature sequence. This ensures that pixels within each group possess relatively consistent gradient patterns during the subsequent pixel grouping stage, allowing the self-attention mechanism to operate more effectively between semantically related pixels. The process is as follows: first, compute the gradient magnitude map m(V) and direction map o(V) of V, then construct the direction-weighted magnitude response R:

(19) $$R=m\left(V\right)⊙\frac{o(V)+\pi }{2\pi }$$

where ⊙ means element-wise multiplication. Compared with relying solely on magnitude, this coupled design more comprehensively reflects the overall consistency of geometric structures. In flat regions, the magnitude is extremely small, so the response approaches zero. In regions with well-defined edges, the magnitude is large and the orientation is distinct, so the response is significantly elevated. In texturally disordered regions, even if the magnitude is relatively high, the low weights from the orientation term effectively suppress the response, thereby distinguishing such regions from true object boundaries. As a differentiable HOG feature encoding operator, this response function provides guidance signals for the subsequent global sorting that are highly correlated with geometric object boundaries.

Summing R over channels for each spatial position yields a scalar importance score, and descending sorting generates indices

(20) $$\mathrm{idx}=\mathrm{Sort}\left(\sum_{c=1}^{C^{\prime }}R_{c}\right).$$

Finally, a unified rearrangement is performed; $R_{C^{\prime }\times H^{\prime }\times W^{\prime }}^{C^{\prime }\times H^{\prime }W^{\prime }}$ denotes flattening the spatial dimensions $H^{\prime }\times W^{\prime }$ of the feature map into a 1D sequence of length $L=H^{\prime }\cdot W^{\prime }$ while keeping the channel dimension $C^{\prime }$ unchanged:

(21) $${\tilde{Q}}_{i}=\mathrm{Gather}\left(R_{C^{\prime }\times H^{\prime }\times W^{\prime }}^{C^{\prime }\times H^{\prime }W^{\prime }}\left(Q_{i}\right),\mathrm{idx}\right),$$

(22) $${\tilde{K}}_{i}=\mathrm{Gather}\left(R_{C^{\prime }\times H^{\prime }\times W^{\prime }}^{C^{\prime }\times H^{\prime }W^{\prime }}\left(K_{i}\right),\mathrm{idx}\right),$$

(23) $$\tilde{V}=\mathrm{Gather}\left(R_{C^{\prime }\times H^{\prime }\times W^{\prime }}^{C^{\prime }\times H^{\prime }W^{\prime }}\left(V\right),\mathrm{idx}\right).$$

**Figure 3:**
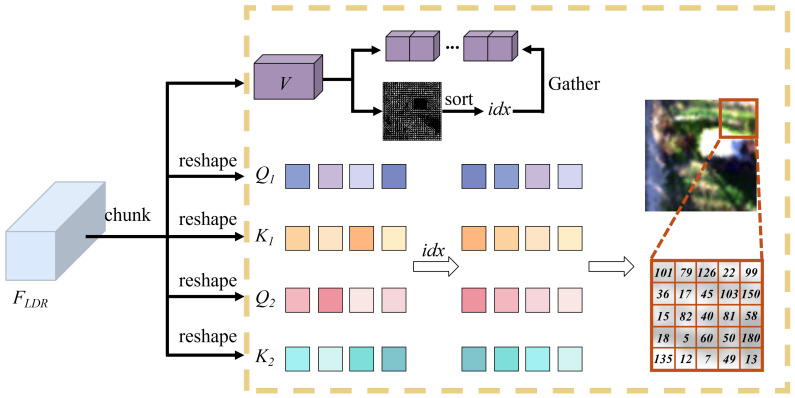
Global sorting: Within the local window marked in the figure, discontinuous indices with large numerical spans appear, indicating that pixel order is no longer confined to local patches but participates in a unified global rearrangement across the entire feature map. All pixels are reordered and clustered globally according to their HOG response scores.

The core of this global sorting operation is to break the rigid constraint of spatial coordinates. Through the direction-weighted magnitude response R constructed in Equation (19), we establish a unified saliency metric that reflects the geometric structure significance of each spatial position. The sorting operation enables pixels that are far apart in the original image but belong to the same semantic class to become adjacent. This provides a highly structured sequential foundation for the subsequent self-attention computation, allowing it to search for semantically relevant points guided by prior information rather than blindly computing global correlations over the cluttered original spatial grid. The process is illustrated in Figure 3.

**(3) Dual-Branch Self-Attention.** To jointly capture both macroscopic features and fine-grained local details, two self-attention branches with different HOG reshaping strategies are introduced in parallel over the sorted features: Bin-based HOG Reshaping (BHOGR) and Frequency-based HOG Reshaping (FHOGR). BHOGR divides the sorted pixels into a fixed number of bins of equal size and computes self-attention independently within each bin. Since the HOG prior reduces the inclusion of pixels with low correlation, pixels within the same bin naturally share similar gradient patterns, allowing this branch to focus on large-scale homogeneous regions such as vast areas of farmland. In contrast, FHOGR performs more sensitive dynamic clustering directly based on HOG response values. Because it is unconstrained by fixed bin sizes, it can more flexibly capture fine textures and local variations such as scattered buildings or roads with sharp edges.

Let $R_{B}\left(\cdot \right)$ and $R_{F}\left(\cdot \right)$ denote the bin-based and frequency-based reshaping operations, respectively. The self-attention computations for the two branches are as follows:

(24) $$A_{B}=\mathrm{Softmax}\left(\frac{R_{B}\left({\tilde{Q}}_{1}\right)R_{B}{\left({\tilde{K}}_{1}\right)}^{⊤}}{\sqrt{d_{k}}}\right)R_{B}\left(\tilde{V}\right)$$

(25) $$A_{F}=\mathrm{Softmax}\left(\frac{R_{F}\left({\tilde{Q}}_{2}\right)R_{F}{\left({\tilde{K}}_{2}\right)}^{⊤}}{\sqrt{d_{k}}}\right)R_{F}\left(\tilde{V}\right)$$

where $d_{k}$ is the dimension scaling factor for the key vectors. The number of attention heads is set to 4, and the fixed bin number in the BHOGR branch is 4. The outputs of the two attention branches are fused via element-wise multiplication:

(26) $$F_{attn}=A_{B}⊙A_{F}.$$

Subsequently, the fused features are restored to their original spatial layout through an inverse sorting operation, and the final output of the DHOGSA module, $F_{DHOGSA}$, is obtained by a 1×1 convolution projection:

(27) $$F_{DHOGSA}={\mathrm{Conv}}_{1\times 1}\left(\mathrm{Undo}\left(F_{attn},\mathrm{idx}\right)\right)$$

where Undo(·,idx) is the inverse operation of Gather.

## 4. Experiments

This section provides the experimental setup and detailed parameters of the proposed MDHANet to ensure reproducibility. Extensive experiments are conducted on four public HSI datasets, and the performance is compared with other models. Ablation studies are also performed to evaluate the contribution of each component to the overall model performance.

### 4.1. Dataset Description

We use four different datasets to evaluate the classification performance of MDHANet. The details of each dataset are provided below.

**Indian Pines (IP) [44]:** This dataset was acquired by the AVIRIS sensor in 1992 over northwestern Indiana, USA. The image size is 145×145 pixels with a spatial resolution of 20 m. The original number of spectral bands is 220; after removing water absorption and low SNR bands, 200 bands are retained, covering the wavelength range of 400–2500 nm. After removing background pixels, 10,249 pixels are selected and classified into 16 classes. The notable characteristic of this dataset is its highly imbalanced class distribution, which poses a significant demand on the classifier’s generalization capability under limited training data.

**WHU-Hi-LongKou (LK) [45]:** This dataset was acquired in 2018 over Longkou town, Hubei province, China, using a Headwall Nano-Hyperspec sensor mounted on an unmanned aerial vehicle (UAV). The study area is a simple agricultural landscape containing nine land cover classes. The UAV flight altitude was 500 m, the image size is 550×400 pixels, the spectral range is 400–1000 nm, and the spatial resolution is approximately 0.463 m. This dataset has a large volume but extremely limited labeled samples, representing a typical few-shot learning scenario that tests the model’s feature extraction capability under an extremely low training ratio.

**WHU-Hi-HongHu (HH) [45]:** This dataset covers Honghu city, Hubei province, China. The image size is 940×475 pixels, consisting of 270 bands in the range 400–1000 nm, with a spatial resolution of approximately 0.043 m. The study area is a complex agricultural scene with a wide variety of crops (22 classes in total). The challenge of this dataset lies in the fact that different cultivars of the same crop exhibit highly similar spectral signatures, making them difficult to distinguish using spectral information alone.

**WHU-Hi-HanChuan (HC) [45]:** The HC dataset was acquired over Hanchuan city, Hubei province, China. It covers an urban–rural fringe area and has 16 classes, 274 spectral bands, and an image size of 1217×303 pixels, with a spatial resolution of 0.109 m. The distinctive characteristic of this dataset is the presence of large shadow-covered regions where object boundaries are obscured, posing a challenge to the classifier’s robustness.

According to the difficulty of the classification task on each dataset, we adopted training ratios of 10%, 0.1%, 1%, and 2% on IP, LK, HH, and HC, respectively. The specific numbers of training and test samples are shown in Table 1.

### 4.2. Experimental Settings and Assessment Criteria

To evaluate the effectiveness of MDHANet, we compared it with several methods, including CNN-based methods, transformer-based methods, and CNN–transformer hybrid methods. The CNN-based methods include: (1) Lite-HCNet [46], a lightweight 2D/3D hybrid convolutional network; (2) LSSAN [47], a lightweight spectral–spatial attention network based on MobileNetV3; and (3) MSDAN [48], which employs a spectral–spatial channel attention mechanism. The transformer-based method is SimPoolFormer [49], which adopts dual-branch feature extraction. The CNN–transformer hybrid method is CACFTNet [50], which integrates covariance attention with cross-layer feature fusion. The overall accuracy (OA), average accuracy (AA), and Kappa coefficient (Kappa) are used to quantitatively evaluate the classification performance of the proposed method compared with other approaches. Experimental results are obtained by averaging five runs. For each method, we report the mean and variance computed over five independent runs rather than the standard deviation. For each metric, higher values indicate better classification performance.

All experiments were implemented with PyTorch 2.3.0 using Python 3.13.12. The hardware environment consisted of a CPU (Intel Xeon Platinum 8352V) and a GPU (NVIDIA GeForce RTX 4090 with 24 GB memory). The number of spectral bands after PCA reduction was 16. The model was trained for 200 epochs using the AdamW optimizer with an initial learning rate of $3\times 10^{-4}$ and a batch size of 512.

### 4.3. Sensitivity Analysis

**Figure 4:**
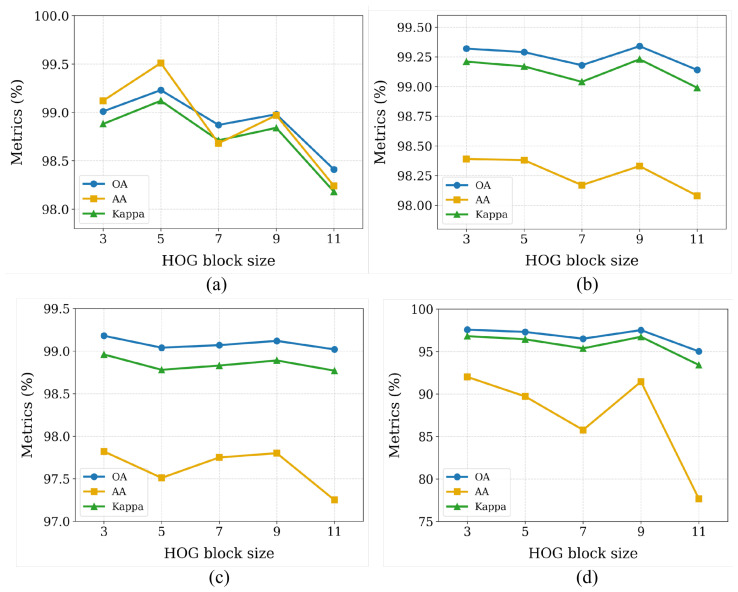
Hyperparameter analysis on different datasets: (**a**) IP (**b**) LK (**c**) HH (**d**) HC.

This section analyzes the influence of the HOG patch size on the classification performance of MDHANet. Experiments are conducted on the IP, LK, HH, and HC datasets, increasing the HOG patch size from 3 to 11 in steps of 2. The results are shown in Figure 4. The optimal value is 5 on IP, 3 on LK, 3 on HH, and 9 on HC. Observing the overall trend, the performance on IP first increases and then decreases with increasing patch size, peaking at 5 and then gradually declining, indicating that a medium local window size provides a good balance of detail preservation and structure perception. On HH, the OA range from size 3 to 11 is only 0.16 percentage points, with minimal fluctuations, demonstrating that MDHANet is insensitive to HOG patch size on HH. HC also exhibits strong stability, achieving its optimum at 9 and then slightly dropping at 11. In contrast, LK is most significantly affected by patch size: as the size increases from 3 to 11, OA drops from 97.58% to 95.01%, and AA plummets from 92.01% to 77.65%. This is because LK has a high spatial resolution; a large HOG patch size introduces many adjacent irrelevant pixels, disrupting the consistency of local gradient statistics and consequently diluting discriminative features.

These results indicate that the choice of HOG patch size is closely related to the spatial resolution and ground-object scale of the dataset. On most datasets, MDHANet maintains stable high accuracy, demonstrating good robustness.

### 4.4. Classification Results Analysis

**Figure 5:**
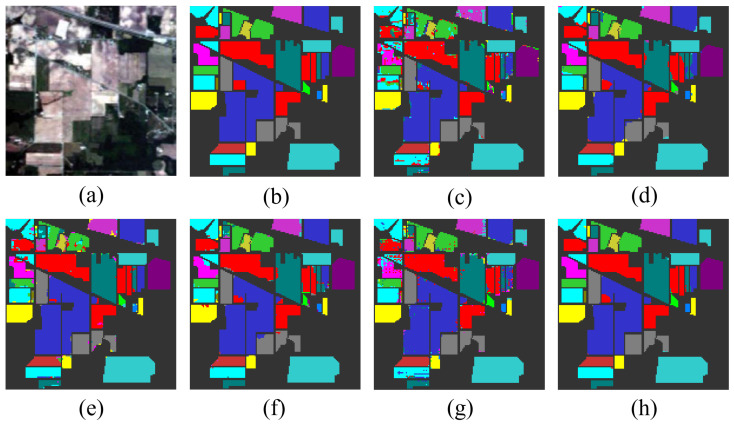
Classification maps obtained by different methods on the IP dataset (sample proportion of 10%): (**a**) false-color image, (**b**) ground-truth map, (**c**) CACFTNet (91.15%), (**d**) Lite-HCNet (98.37%), (**e**) LSSAN (93.92%), (**f**) MSDAN (96.39%), (**g**) SimPoolFormer (92.38%), (**h**) MDHANet (99.23%).

**Figure 6:**
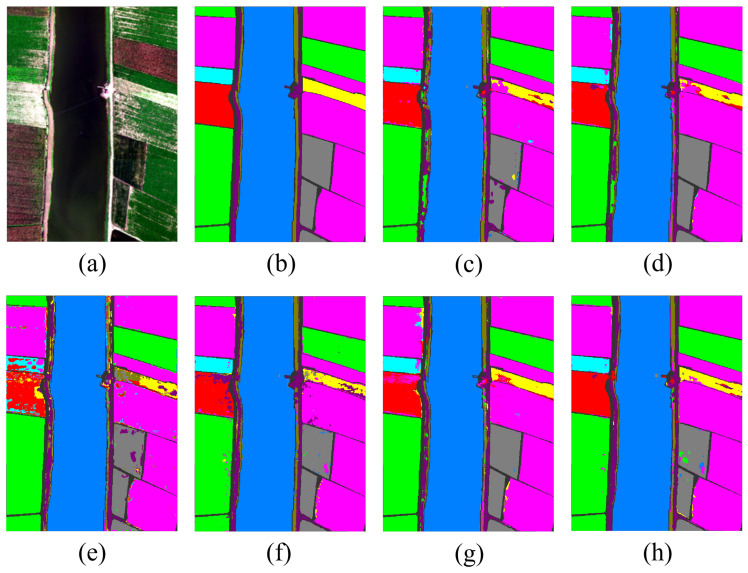
Classification maps obtained by different methods on the LK dataset (sample proportion of 0.1%): (**a**) false-color image, (**b**) ground-truth map, (**c**) CACFTNet (96.55%), (**d**) Lite-HCNet (97.04%), (**e**) LSSAN (93.47%), (**f**) MSDAN (96.68%), (**g**) SimPoolFormer (96.79%), (**h**) MDHANet (97.58%).

**Figure 7:**
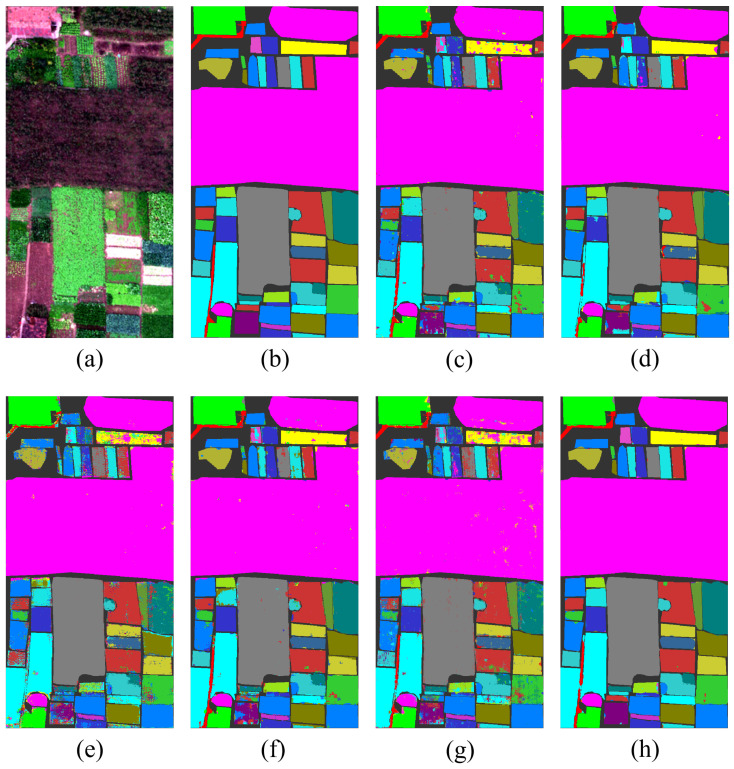
Classification maps obtained by different methods on the HH dataset (sample proportion of 1%): (**a**) false-color image, (**b**) ground-truth map, (**c**) CACFTNet (97.02%), (**d**) Lite-HCNet (96.95%), (**e**) LSSAN (90.47%), (**f**) MSDAN (95.85%), (**g**) SimPoolFormer (93.07%), (**h**) MDHANet (99.18%).

**Figure 8:**
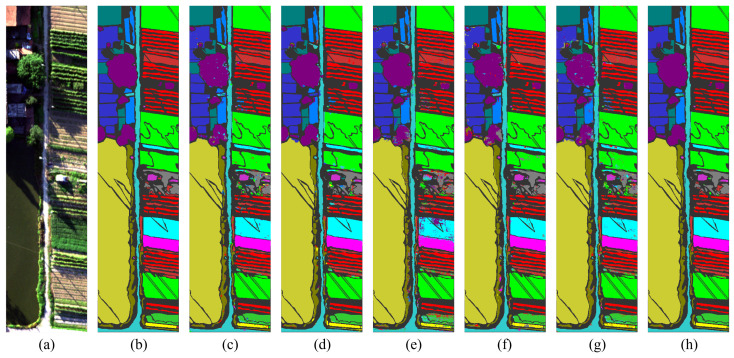
Classification maps obtained by different methods on the HC dataset (Sample proportion of 2%): (**a**) false-color image, (**b**) ground-truth map, (**c**) CACFTNet (96.89%), (**d**) Lite-HCNet (96.98%), (**e**) LSSAN (93.08%), (**f**) MSDAN (96.74%), (**g**) SimPoolFormer (94.83%), (**h**) MDHANet (99.34%).

Table 2, Table 3, Table 4 and Table 5 summarize the classification results obtained on the IP, LK, HH, and HC datasets, with the best results shown in bold. Figure 5, Figure 6, Figure 7 and Figure 8 also provide intuitive visualizations of the classification maps.

**(1) Results On IP.**

Table 2 summarizes the classification accuracy of each method on the IP dataset. It can be observed that the proposed MDHANet achieves the best results across all evaluation metrics: OA reaches 99.13%, AA 99.12%, and Kappa 99.01%. Compared with the second-best method (Lite-HCNet), it improves OA by 0.83%, and both AA and Kappa by 0.95%. Moreover, four land cover classes (“Alfalfa”, “Hay-windrowed”, “Oats”, “Building-grass-trees-drives”) achieve 100% accuracy, and other classes also obtain satisfactory results. These results fully demonstrate that MDHANet maintains extremely high classification accuracy and stability even under class imbalance, establishing it as an effective and robust HSI classifier.

From Figure 5, it can be observed that the classification map of MDHANet is smooth overall and achieves the best visual quality, with clear object contours and no obvious misclassification regions. The classification maps of CACFTNet, LSSAN, and SimPoolFormer are generally noisy, with large areas of misclassification in some regions, while Lite-HCNet and MSDAN produce relatively smoother maps but still contain some noise in the upper left corner. For the “Oats” class, which has very few training samples, most compared models misclassify it as surrounding categories, whereas MDHANet successfully identifies these small patches from a rare class. The above discussion intuitively shows that reusing feature across different scales enables MDHANet to maintain strongly discriminative feature representations even under uneven data distribution.

**(2) Results On LK.**

Table 3 presents the classification results on the Longkou dataset. MDHANet achieves an OA of 97.32%, AA of 90.69%, and Kappa of 96.46%, outperforming the second-best method by 0.41%, 1.69%, and 0.53%, respectively. Lite-HCNet achieves the best results on the “Corn” and “Rice” classes, while SimPoolFormer obtains the best on “Narrow-leaf soybean”; however, the proposed method maintains the highest overall accuracy and achieves significant improvements on certain difficult classes, such as a 4.84% improvement over MSDAN on “Roads and houses”. This improvement is attributed to our feature rearrangement strategy, which guides the model to focus on globally similar pixels. The data in the table indicate that even in challenging scenarios with extremely limited training samples (e.g., several crop classes such as “Sesame” with no more than ten training samples each), MDHANet maintains superior classification capability and stability.

As shown in Figure 6, the MDHANet classification map produces the least noise overall. CACFTNet, Lite-HCNet, and LSSAN perform poorly on the strip-like “Roads and houses” and “Mixed weed” on both sides, impairing the connectivity of the complete structures. MSDAN and SimPoolFormer generate speckle noise within the cotton planting area. In contrast, MDHANet, with its large-scale receptive field and HOG-driven global sorting strategy, preserves the structural integrity of linear objects and achieves higher purity within regions.

**(3) Results On HH.**

Table 4 summarizes the classification results on the Honghu dataset. The proposed MDHANet achieves an OA of 99.02%, an AA of 97.65%, and a Kappa of 98.76%, surpassing the second-best method CACFTNet by 2.69%, 7.47%, and 3.40%, respectively. HH contains easily confusable categories that have similar spectral characteristics but belong to different fields. Due to extremely weak inter-class differences, most compared methods struggle to effectively distinguish these categories. The HOG prior used by MDHANet enhances the perception of field boundaries and spatial structures, helping it to achieve significant improvements on easily confused categories thanks to the use of explicit spatial partitioning. Example results include 98.87% on “Chinese cabbage” (3.52% improvement over the second-best), 96.17% on “Brassica chinensis” (10.02% improvement), and 97.92% on “Small Brassica chinensis” (all other methods below 91%). These results demonstrate that MDHANet can effectively overcome misclassification caused by spectral confusion, exhibiting excellent classification accuracy and robustness in complex agricultural scenes.

From Figure 7, the classification map of MDHANet shows the highest consistency with the ground truth map and more regular object boundaries. LSSAN and SimPoolFormer exhibit large areas of scattered noise, while CACFTNet, Lite-HCNet, and MSDAN all show obvious confusion between “Brassica chinensis” and “Small Brassica chinensis”, likely due to their lack of structural priors. In contrast, our DHOGSA module enables the model to accurately classify pixels even with similar spectral information based on their spatial positions.

**(4) Results On HC.**

Table 5 summarizes the classification results on the Hanchuan dataset. MDHANet achieves 99.24% OA, 98.09% AA, and 99.12% Kappa, improving over the second-best method (CACFTNet) by 2.47%, 4.50%, and 2.91%, respectively. The HC dataset contains large shadowed areas due to low-elevation sunlight, which obscures object edges. The “Watermelon” class, which is particularly affected by shadow coverage, poses a considerable challenge for most methods, with the second-best method attaining only 86.99% while MDHANet achieves 96.10%. On “Water spinach”, MDHANet achieves 100.00%, while on “Cowpea” it outperforms the second-best model by 2.77%. These improvements are attributed to the MDFE module that effectively preserves and reuses edge information in shadowed areas, allowing our method to maintain high accuracy and strong robustness under shadow interference.

As shown in Figure 8, MDHANet produces the classification map that is most similar to the ground truth, while all other compared methods exhibit varying degrees of confusion and noise. Taking the complex region formed by “Watermelon”, “Bright object”, and “Bare soil” as an example, CACFTNet, LSSAN, and SimPoolFormer all generate a large amount of misclassification noise within this region, causing fragmentation. Lite-HCNet and MSDAN both produce relatively smoother overall maps, but still have certain errors at the boundaries of each class. In contrast, our MDHANet maintains accurate and clear class boundaries. For the slender and curved strip-shaped area planted with grass, all of the compared methods produce local noise, while MDHANet correctly preserves the complete structure. This is attributed to the global context provided by the large-scale branch and the HOG-driven pixel grouping strategy. These positive results demonstrate the high effectiveness of the information flow control strategy of multi-scale feature reuse and fusion in our method.

### 4.5. Ablation Study

To validate the effectiveness of the DCU and the DHOGSA module in MDHANet, we conduct ablation experiments on the four datasets. Starting from a baseline, we gradually add the DCU and the DHOGSA module, designing four configurations: Baseline, Baseline + DCU, Baseline + DHOGSA, and Baseline + DCU + DHOGSA (the complete MDHANet). The experimental results are shown in Table 6, with the best results in bold.

As shown in Table 6, when the DCU is introduced alone, the OA on the four datasets improves by 0.28%, 0.13%, 2.08%, and 1.51%, respectively. The gains are more significant on complex datasets such as HH and HC, confirming the effectiveness of the multi-scale feature reuse mechanism in mitigating detail information loss. Introducing the DHOGSA module alone also improves model performance. The OA improvements on HH and HC are as high as 6.62% and 4.17%, respectively. This is highly consistent with the intrinsic characteristics of the datasets. HH contains many spectrally similar crops belonging to different fields, while HC has large shadows due to low-elevation illumination, disrupting edge continuity. DHOGSA explicitly encodes edge contours and gradient distributions using HOG. This module clusters pixels of homogeneous regions through local intra-patch rearrangement and global sorting, effectively alleviating misclassification caused by spectral confusion and shadow edges. When DCU and DHOGSA are used together, the model achieves the best performance on all datasets. Taking HC as an example, the complete MDHANet achieves 99.24% OA, 98.09% AA, and 99.12% Kappa, all higher than using either module alone. This is because the two modules are complementary: DCU provides a rich feature basis for DHOGSA through multi-scale cross-layer feature reuse, while DHOGSA explicitly models spatial structures based on the HOG prior, compensating for the deficiency of pure data-driven methods in perceiving gradient patterns. The above ablation results fully demonstrate the complementary effectiveness of the two modules and explain the intrinsic reason why the complete MDHANet achieves the best performance on all four datasets.

**Figure 9:**
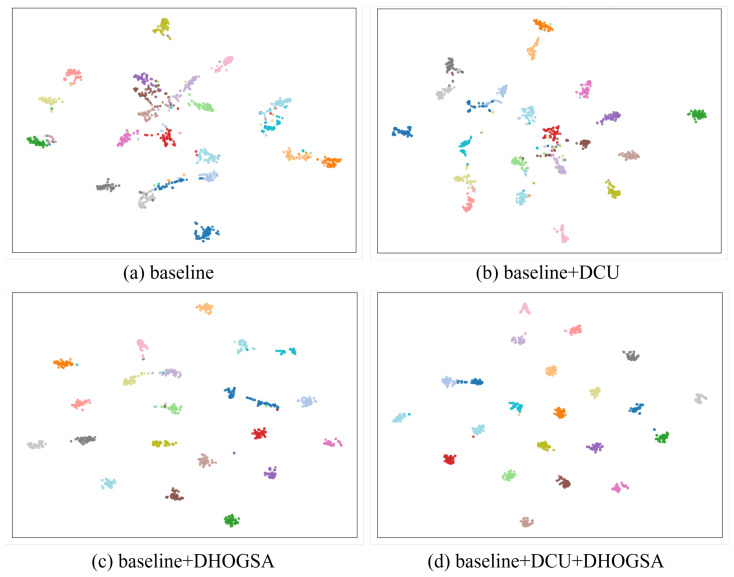
t-SNE visualization of feature distributions on the HH dataset under different module configurations.

To further verify the effectiveness of the HOG mechanism in enhancing spatial representation and improving the separability of classes with similar spectral signatures, we extracted deep features from the network under different module configurations and performed t-SNE visualization on the HH dataset. It can be clearly observed from Figure 9 that for the network relying solely on implicit feature learning (e.g., Figure 9a,b), several of the classes exhibit severe overlap and confusion in the feature space when processing HSI data. After introducing the HOG mechanism as a spatial structure prior (Figure 9c,d), the distribution in the feature space is significantly improved; specifically, the inter-class separation is notably enlarged, while the intra-class compactness is enhanced. This demonstrates that the HOG mechanism successfully injects strong spatial discriminative information for spectrally similar classes by guiding the network to focus on local gradient orientations and the edge geometries of ground objects.

### 4.6. Computational Cost Analysis

To evaluate the practical deployability of the proposed MDHANet, we report its computational cost on the four benchmark datasets in terms of model parameters (Params), floating-point operations (FLOPs), and per-sample inference time. FLOPs are calculated using the thop library, and inference time is averaged over the entire test set on a single NVIDIA GeForce RTX 4090 GPU.

As shown in Table 7, the parameter count of MDHANet is determined solely by the network architecture, and the moderate size (3.5 M) makes it feasible for deployment. FLOPs vary with the input spatial resolution: datasets with larger patches (IP, HH, HC) require approximately 3.72G FLOPs, while LK with a smaller patch requires 0.84G FLOPs. In terms of inference speed, MDHANet achieves stable per-sample inference times ranging from 13.61 ms to 14.18 ms across the four datasets. In summary, MDHANet strikes a favorable balance between accuracy and efficiency, providing a practical and effective solution for real-world applications such as agricultural monitoring and urban land cover mapping.

## 5. Conclusions

Aiming to address limitations of existing hyperspectral image classification methods such as prominent gradient vanishing, information loss, and failure to effectively utilize explicit spatial structure information, this paper proposes MDHANet, a multi-scale dense self-attention network incorporating HOG priors. This method employs three parallel densely connected branches at different scales, enabling efficient information flow between shallow and deep layers that effectively alleviates gradient vanishing while maximizing feature reuse. Second, we design a dynamic HOG-driven self-attention module (DHOGSA) that incorporates HOG into the self-attention computation as a learnable prior. This module first uses local dynamic range convolution (LDRConv) to reorganize the pixel order within local windows according to gradient distributions, enabling convolution kernels to operate on sets of pixels with consistent structures. Then, a HOG-driven global sorting strategy aggregates spatially dispersed pixels with similar gradient patterns in the feature dimension. Finally, dual-branch self-attention (bin-based and frequency-based) models large-area homogeneous regions and fine-grained textures, respectively. These designs endow the model with powerful perception and discrimination capabilities for spatial structure information of ground objects.

Experimental results on four public HSI datasets show that MDHANet significantly outperforms various state-of-the-art methods in terms of OA, AA, and Kappa. Ablation studies further validate the complementary effectiveness of the dense connection unit and the DHOGSA module. Nevertheless, the proposed method mainly relies on HOG as a spatial structure prior. Improving the generalization capability of the model by incorporating additional types of prior knowledge such as spectral priors or multimodal information will be a focus of our future work.

## Figures and Tables

**Table 1:** Number of training and test samples for the IP, LK, HH, and HC datasets.

IP			LK		
Class	Train	Test	Class	Train	Test
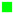 Alfalfa	5	39	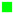 Corn	35	34,476
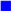 Corn-notill	143	1285	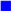 Cotton	9	8365
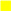 Corn-mintill	83	747	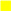 Sesame	4	3027
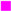 Corn	24	213	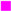 Broad-leaf soybean	64	63,148
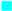 Grass-pasture	49	434	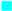 Narrow-leaf soybean	5	4146
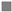 Grass-trees	73	657	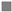 Rice	12	11,842
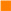 Grass-pasture-mowed	3	25	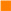 Water	68	66,988
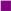 Hay-windrowed	48	430	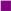 Roads and houses	8	7116
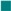 Oats	2	18	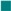 Mixed weed	6	5223
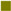 Soybean-notill	98	874			
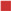 Soybean-mintill	246	2209			
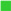 Soybean-clean	60	533			
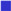 Wheat	21	184			
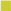 Woods	127	1138			
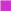 Building-grass-trees-drives	39	347			
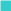 Stone-steel-towers	10	83			
**HH**			**HC**		
**Class**	**train**	**test**	**Class**	**train**	**test**
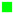 Red roof	141	13,900	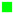 Strawberry	895	43,840
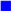 Road	36	3476	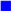 Cowpea	456	22,297
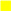 Bare soil	219	21,602	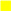 Soybean	206	10,081
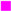 Cotton	1633	161,652	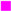 Sorghum	108	5245
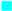 Cotton firewood	63	6155	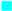 Water spinach	24	1176
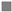 Rape	446	44,111	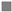 Watermelon	91	4442
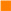 Chinese cabbage	242	23,861	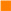 Greens	119	5784
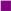 Pakchoi	41	4013	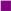 Trees	360	17,618
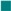 Cabbage	109	10,710	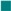 Grass	190	9279
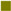 Tuber mustard	124	12,270	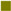 Red roof	211	10,305
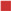 Brassica parochinensis	111	10,904	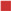 Gray roof	339	16,572
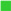 Brassica chinensis	90	8864	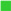 Plastic	74	3605
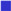 Small Brassica chinensis	226	22,281	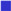 Bare soil	183	8933
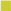 Lactuca sativa	74	7282	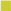 Road	372	18,188
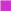 Celtuce	11	991	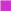 Bright object	23	1113
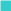 Film covered lettuce	73	7189	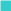 Water	1509	73,892
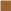 Romaine lettuce	31	2979			
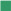 Carrot	33	3184			
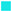 White radish	88	8624			
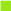 Garlic sprout	35	3451			
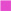 Broad bean	14	1314			
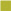 Tree	41	3999			

**Table 2:** Classification results on the IP dataset using 10% training samples (%).

Class	CACFTNet	Lite-HCNet	LSSAN	MSDAN	SimPoolFormer	MDHANet
Alfalfa	34.15 ± 0.00	98.54 ± 3.81	76.59 ± 34.77	64.39 ± 27.57	98.05 ± 15.24	**100.00 ± 0.00**
Corn-notill	89.18 ± 0.69	94.57 ± 0.16	91.72 ± 0.34	93.88 ± 0.23	87.22 ± 0.20	**97.07 ± 0.05**
Corn-mintill	78.95 ± 0.80	99.17 ± 0.47	87.09 ± 23.81	97.72 ± 0.26	86.77 ± 0.31	**99.41 ± 0.10**
Corn	60.75 ± 2.53	98.68 ± 0.57	78.31 ± 15.73	92.49 ± 14.10	68.82 ± 7.63	**99.91 ± 0.04**
Grass-pasture	94.76 ± 0.81	99.03 ± 0.45	93.52 ± 4.54	98.21 ± 1.55	93.56 ± 0.91	**99.63 ± 0.10**
Grass-trees	98.05 ± 0.12	99.33 ± 0.05	96.96 ± 0.55	97.50 ± 2.59	97.96 ± 0.34	**99.12 ± 0.18**
Grass-pasture-mowed	63.20 ± 2.56	99.20 ± 2.56	86.40 ± 125.44	84.80 ± 47.36	93.60 ± 10.24	**100.00 ± 0.00**
Hay-windrowed	96.46 ± 0.07	99.81 ± 0.14	99.72 ± 0.12	99.77 ± 0.04	**100.00 ± 0.00**	**100.00 ± 0.00**
Oats	31.11 ± 44.45	93.33 ± 4.93	73.33 ± 177.73	54.44 ± 190.15	40.00 ± 4.93	**97.78 ± 7.42**
Soybean-notill	91.66 ± 0.04	98.01 ± 1.19	93.40 ± 1.25	95.96 ± 0.03	88.89 ± 0.13	**98.68 ± 0.07**
Soybean-mintill	95.94 ± 0.04	98.89 ± 0.17	98.05 ± 0.80	96.89 ± 1.34	94.02 ± 0.07	**99.61 ± 0.00**
Soybean-clean	82.17 ± 2.24	96.82 ± 3.21	80.64 ± 16.60	90.19 ± 16.52	88.43 ± 0.98	**98.99 ± 0.29**
Wheat	98.16 ± 0.54	99.03 ± 1.10	95.67 ± 5.15	98.92 ± 0.35	98.81 ± 0.75	**99.68 ± 0.42**
Woods	99.17 ± 0.01	99.95 ± 0.00	99.47 ± 0.09	99.81 ± 0.04	98.61 ± 0.12	**100.00 ± 0.00**
Building-grass-trees-drives	78.33 ± 0.51	99.02 ± 0.75	89.57 ± 3.96	92.74 ± 4.63	85.59 ± 0.83	**99.71 ± 0.00**
Stone-steel-towers	75.71 ± 1.47	97.38 ± 12.12	70.95 ± 17.90	84.29 ± 11.02	94.05 ± 0.57	**96.43 ± 2.27**
OA	90.83 ± 0.07	98.30 ± 0.01	93.55 ± 0.25	96.07 ± 0.09	91.89 ± 0.10	**99.13 ± 0.01**
AA	79.24 ± 0.26	98.17 ± 0.08	88.21 ± 0.64	90.12 ± 1.62	88.40 ± 0.36	**99.12 ± 0.05**
Kappa	89.53 ± 0.09	98.06 ± 0.02	92.61 ± 0.34	95.52 ± 0.11	90.74 ± 0.13	**99.01 ± 0.01**

**Table 3:** Classification results on the LK dataset using 0.1% training samples (%).

Class	CACFTNet	Lite-HCNet	LSSAN	MSDAN	SimPoolFormer	MDHANet
Corn	99.67 ± 0.00	**99.97 ± 0.00**	99.73 ± 0.03	97.88 ± 0.21	99.79 ± 0.01	99.60 ± 0.10
Cotton	**96.06 ± 0.37**	95.15 ± 11.16	79.62 ± 23.28	89.01 ± 17.32	82.96 ± 0.19	**96.33 ± 1.38**
Sesame	89.31 ± 8.88	**94.22 ± 6.40**	35.13 ± 352.38	93.84 ± 1.45	91.13 ± 2.33	92.43 ± 8.22
Broad-leaf soybean	**99.66 ± 0.01**	98.88 ± 0.08	96.76 ± 0.88	99.33 ± 0.09	98.34 ± 0.02	**99.66 ± 0.00**
Narrow-leaf soybean	69.88 ± 30.44	64.94 ± 18.42	33.15 ± 247.86	56.07 ± 75.62	**82.14 ± 16.22**	78.28 ± 41.07
Rice	93.30 ± 2.19	**99.37 ± 0.28**	90.49 ± 1.92	92.47 ± 8.23	99.33 ± 0.07	93.49 ± 0.46
Water	99.04 ± 0.05	99.81 ± 0.00	**99.89 ± 0.03**	99.85 ± 0.02	99.76 ± 0.01	99.19 ± 0.33
Roads and houses	63.85 ± 11.47	80.64 ± 64.24	75.43 ± 81.90	87.81 ± 14.80	85.07 ± 1.29	**92.65 ± 2.84**
Mixed weed	74.04 ± 3.88	62.14 ± 23.87	34.01 ± 22.56	**76.71 ± 16.59**	62.46 ± 1.53	64.60 ± 30.05
OA	96.28 ± 0.03	96.91 ± 0.09	92.67 ± 0.37	96.50 ± 0.02	96.66 ± 0.01	**97.32 ± 0.01**
AA	87.20 ± 0.33	88.35 ± 1.04	71.58 ± 19.20	88.11 ± 1.44	89.00 ± 0.38	**90.69 ± 0.74**
Kappa	95.10 ± 0.05	95.93 ± 0.16	90.35 ± 0.66	95.38 ± 0.03	95.60 ± 0.02	**96.46 ± 0.02**

**Table 4:** Classification results on the HH dataset using 1% training samples (%).

Class	CACFTNet	Lite-HCNet	LSSAN	MSDAN	SimPoolFormer	MDHANet
Red roof	97.41 ± 0.48	97.25 ± 1.30	97.24 ± 0.53	97.17 ± 1.11	96.26 ± 0.00	**98.83 ± 0.10**
Road	80.54 ± 39.75	65.09 ± 317.98	50.73 ± 378.21	78.22 ± 104.93	79.34 ± 0.09	**91.51 ± 1.87**
Bare soil	96.03 ± 2.39	96.67 ± 7.30	92.53 ± 1.91	97.28 ± 1.42	93.77 ± 0.00	**98.32 ± 0.11**
Cotton	99.70 ± 0.00	99.86 ± 0.01	99.56 ± 0.00	99.54 ± 0.15	99.12 ± 0.00	**99.95 ± 0.00**
Cotton firewood	88.21 ± 26.23	88.82 ± 71.97	69.86 ± 166.68	91.80 ± 8.61	69.25 ± 0.05	**99.64 ± 0.08**
Rape	98.90 ± 0.03	99.25 ± 0.16	98.08 ± 0.04	98.77 ± 0.98	96.14 ± 0.00	**99.83 ± 0.00**
Chinese cabbage	95.03 ± 0.22	95.35 ± 0.43	89.74 ± 10.30	94.53 ± 4.86	90.83 ± 0.02	**98.87 ± 0.01**
Pakchoi	78.98 ± 4.84	84.49 ± 12.12	49.08 ± 67.25	68.23 ± 213.08	66.29 ± 0.11	**97.62 ± 0.89**
Cabbage	98.58 ± 0.46	99.37 ± 0.09	88.87 ± 28.65	98.03 ± 3.20	98.06 ± 0.00	**99.55 ± 0.04**
Tuber mustard	93.71 ± 0.83	93.92 ± 2.16	82.32 ± 43.42	94.53 ± 3.48	85.40 ± 0.00	**98.34 ± 0.01**
Brassica parochinensis	88.48 ± 0.11	89.28 ± 2.81	75.71 ± 90.87	83.92 ± 6.97	81.93 ± 0.05	**96.13 ± 0.20**
Brassica chinensis	86.15 ± 3.67	85.33 ± 37.09	80.11 ± 29.55	74.37 ± 73.29	70.57 ± 0.22	**96.17 ± 0.19**
Small Brassica chinensis	90.96 ± 6.14	90.74 ± 29.75	85.40 ± 14.14	88.67 ± 13.20	81.45 ± 0.02	**97.92 ± 0.07**
Lactuca sativa	94.16 ± 4.97	94.54 ± 8.35	72.28 ± 66.40	95.36 ± 3.09	84.97 ± 0.05	**98.47 ± 0.29**
Celtuce	84.63 ± 6.20	88.63 ± 28.89	75.32 ± 75.97	82.13 ± 95.54	83.84 ± 0.03	**93.59 ± 0.14**
Film covered lettuce	98.66 ± 0.13	95.33 ± 7.77	91.95 ± 2.03	90.80 ± 48.65	93.62 ± 0.01	**98.76 ± 0.06**
Romaine lettuce	90.60 ± 2.27	93.88 ± 13.04	84.08 ± 53.93	88.93 ± 131.37	91.43 ± 0.01	**99.03 ± 0.27**
Carrot	93.80 ± 1.81	89.35 ± 65.19	77.22 ± 89.28	74.41 ± 308.73	92.46 ± 0.01	**96.28 ± 0.24**
White radish	92.28 ± 1.64	91.94 ± 4.69	72.27 ± 1.58	86.20 ± 58.97	92.26 ± 0.03	**97.12 ± 1.31**
Garlic sprout	87.15 ± 8.50	87.89 ± 14.02	47.34 ± 540.83	80.43 ± 209.76	81.34 ± 0.09	**94.13 ± 0.66**
Broad bean	52.80 ± 453.86	36.65 ± 403.52	22.16 ± 366.46	28.55 ± 663.65	38.64 ± 0.54	**99.46 ± 0.06**
Tree	97.22 ± 1.83	92.77 ± 41.17	85.91 ± 38.16	88.05 ± 35.85	88.06 ± 0.04	**98.79 ± 0.12**
OA	96.33 ± 0.13	96.28 ± 0.35	91.52 ± 5.82	95.00 ± 0.43	93.04 ± 0.00	**99.02 ± 0.00**
AA	90.18 ± 0.40	88.93 ± 1.42	76.72 ± 51.13	85.45 ± 5.57	84.32 ± 0.01	**97.65 ± 0.01**
Kappa	95.36 ± 0.21	95.29 ± 0.57	89.25 ± 9.38	93.67 ± 0.68	91.19 ± 0.00	**98.76 ± 0.01**

**Table 5:** Classification results on the HC dataset using 2% training samples (%).

Class	CACFTNet	Lite-HCNet	LSSAN	MSDAN	SimPoolFormer	MDHANet
Strawberry	98.82 ± 0.08	98.72 ± 0.25	98.60 ± 0.83	98.71 ± 0.09	97.73 ± 0.00	**99.80 ± 0.00**
Cowpea	94.56 ± 0.06	95.46 ± 3.43	93.72 ± 1.11	96.67 ± 1.74	93.09 ± 0.01	**99.44 ± 0.03**
Soybean	97.84 ± 0.15	97.90 ± 0.82	90.32 ± 20.62	95.49 ± 4.88	94.95 ± 0.24	**99.89 ± 0.00**
Sorghum	98.92 ± 0.01	99.44 ± 0.28	95.10 ± 5.31	97.58 ± 5.05	98.35 ± 0.02	**99.51 ± 0.02**
Water spinach	90.30 ± 10.61	95.15 ± 27.08	78.41 ± 117.07	74.80 ± 666.36	75.15 ± 0.73	**100.00 ± 0.00**
Watermelon	77.78 ± 2.89	86.99 ± 12.35	58.51 ± 17.80	73.00 ± 387.04	71.11 ± 0.57	**96.10 ± 0.14**
Greens	94.26 ± 0.42	91.08 ± 19.75	88.05 ± 7.91	86.54 ± 26.83	89.30 ± 0.07	**98.46 ± 0.00**
Trees	94.45 ± 1.31	93.91 ± 7.41	87.95 ± 5.15	89.33 ± 10.22	90.97 ± 0.10	**99.00 ± 0.01**
Grass	93.62 ± 0.57	92.33 ± 2.34	79.69 ± 21.99	87.01 ± 1.86	92.20 ± 0.04	**97.62 ± 0.30**
Red roof	98.78 ± 0.27	97.07 ± 9.52	93.86 ± 5.67	96.60 ± 2.56	98.80 ± 0.01	**99.15 ± 0.02**
Gray roof	98.60 ± 0.28	96.21 ± 6.33	96.50 ± 2.14	97.10 ± 3.37	96.59 ± 0.00	**99.64 ± 0.01**
Plastic	91.55 ± 0.57	88.84 ± 32.61	55.56 ± 51.09	74.63 ± 260.64	79.77 ± 8.75	**99.10 ± 0.15**
Bare soil	84.40 ± 0.58	79.13 ± 9.56	70.08 ± 12.36	82.18 ± 14.41	74.60 ± 0.05	**94.21 ± 0.14**
Road	95.60 ± 0.16	94.83 ± 2.60	91.15 ± 4.24	94.56 ± 2.36	92.77 ± 0.01	**99.18 ± 0.01**
Bright object	88.18 ± 0.67	77.69 ± 15.41	25.79 ± 37.38	56.50 ± 1277.11	73.11 ± 2.59	**88.42 ± 1.14**
Water	99.76 ± 0.00	99.84 ± 0.02	99.75 ± 0.03	99.61 ± 0.11	99.55 ± 0.00	**99.96 ± 0.00**
OA	96.77 ± 0.02	96.33 ± 0.26	92.81 ± 0.11	95.11 ± 2.06	94.74 ± 0.01	**99.24 ± 0.00**
AA	93.59 ± 0.05	92.79 ± 0.77	81.44 ± 0.30	87.52 ± 37.00	88.63 ± 0.05	**98.09 ± 0.02**
Kappa	96.21 ± 0.02	95.70 ± 0.35	91.57 ± 0.16	94.27 ± 2.83	93.84 ± 0.01	**99.12 ± 0.00**

**Table 6:** Results of the ablation study on the four datasets.

Dataset	Baseline	DCU	DHOGSA	OA (%)	AA (%)	Kappa (%)
IP	*√*			98.70	92.96	98.52
	*√*	*√*		98.98	98.09	98.84
	*√*		*√*	99.02	**99.21**	98.89
	*√*	*√*	*√*	**99.13**	99.12	**99.01**
LK	*√*			97.10	92.28	96.19
	*√*	*√*		97.23	**92.67**	96.35
	*√*		*√*	97.18	89.95	96.26
	*√*	*√*	*√*	**97.32**	90.69	**96.46**
HH	*√*			92.04	84.29	89.92
	*√*	*√*		94.12	87.86	92.56
	*√*		*√*	98.66	96.19	98.31
	*√*	*√*	*√*	**99.02**	**97.65**	**98.76**
HC	*√*			94.79	90.63	93.90
	*√*	*√*		96.30	92.64	95.66
	*√*		*√*	98.96	97.92	98.78
	*√*	*√*	*√*	**99.24**	**98.09**	**99.12**

**Table 7:** Computational cost of MDHANet on the four datasets.

Dataset	Params (M)	FLOPs (G)	Inference (ms)
IP	3.50	3.7215	13.78
LK	3.50	0.8351	14.18
HH	3.50	3.7215	13.73
HC	3.50	3.7215	13.61

## Data Availability

The Indian Pines dataset is available at: https://purr.purdue.edu/publications/1947/ (accessed on 1 April 2026). The WHU-Hi datasets (Longkou, Honghu, and Hanchuan) are available at: https://rsidea.whu.edu.cn/resource_WHUHi_sharing.htm (accessed on 1 April 2026).

## References

[1] Li N., Wang Z., Cheikh F.A. (2024). Discriminating Spectral–Spatial Feature Extraction for Hyperspectral Image Classification: A Review. Sensors.

[2] Lu G., Fei B. (2014). Medical hyperspectral imaging: A review. J. Biomed. Opt..

[3] Rasti B., Hong D., Hang R., Ghamisi P., Kang X., Chanussot J., Benediktsson J.A. (2020). Feature Extraction for Hyperspectral Imagery: The Evolution From Shallow to Deep: Overview and Toolbox. IEEE Geosci. Remote Sens. Mag..

[4] Zhao Y., Zhang Y. (2026). Applications of Deep Learning in UAV-Based Hyperspectral Remote Sensing: A Review. Remote Sens..

[5] Ghamisi P., Plaza J., Chen Y., Li J., Plaza A.J. (2017). Advanced Spectral Classifiers for Hyperspectral Images: A review. IEEE Geosci. Remote Sens. Mag..

[6] Liu J., Feng Q., Liang T., Yin J., Gao J., Ge J., Hou M., Wu C., Li W. (2022). Estimating the Forage Neutral Detergent Fiber Content of Alpine Grassland in the Tibetan Plateau Using Hyperspectral Data and Machine Learning Algorithms. IEEE Trans. Geosci. Remote Sens..

[7] Guo J., Hong D., Zhu X.X. (2024). High-resolution satellite images reveal the prevalent positive indirect impact of urbanization on urban tree canopy coverage in South America. Landsc. Urban Plan..

[8] Rogge D.M., Rivard B., Zhang J., Feng J. (2006). Iterative Spectral Unmixing for Optimizing Per-Pixel Endmember Sets. IEEE Trans. Geosci. Remote Sens..

[9] Davies B.F.R., Gernez P., Geraud A., Oiry S., Rosa P., Zoffoli M.L., Barillé L. (2023). Multi- and hyperspectral classification of soft-bottom intertidal vegetation using a spectral library for coastal biodiversity remote sensing. Remote Sens. Environ..

[10] Gualtieri J.A., Chettri S. Support vector machines for classification of hyperspectral data. Proceedings of the 2000 IEEE International Geoscience and Remote Sensing Symposium (IGARSS).

[11] Mercier G., Lennon M. Support vector machines for hyperspectral image classification with spectral-based kernels. Proceedings of the 2003 IEEE International Geoscience and Remote Sensing Symposium (IGARSS).

[12] Melgani F., Bruzzone L. (2004). Classification of hyperspectral remote sensing images with support vector machines. IEEE Trans. Geosci. Remote Sens..

[13] Ham J., Chen Y., Crawford M.M., Ghosh J. (2005). Investigation of the random forest framework for classification of hyperspectral data. IEEE Trans. Geosci. Remote Sens..

[14] Zhang Y., Cao G., Li X., Wang B. (2018). Cascaded Random Forest for Hyperspectral Image Classification. IEEE J. Sel. Top. Appl. Earth Obs. Remote Sens..

[15] Li J., Bioucas-Dias J.M., Plaza A. (2010). Semisupervised Hyperspectral Image Segmentation Using Multinomial Logistic Regression with Active Learning. IEEE Trans. Geosci. Remote Sens..

[16] Li J., Bioucas-Dias J.M., Plaza A. (2012). Spectral–Spatial Hyperspectral Image Segmentation Using Subspace Multinomial Logistic Regression and Markov Random Fields. IEEE Trans. Geosci. Remote Sens..

[17] Cariou C., Chehdi K. A new k-nearest neighbor density-based clustering method and its application to hyperspectral images. Proceedings of the 2016 IEEE International Geoscience and Remote Sensing Symposium (IGARSS).

[18] Li F., Lu H. Hyperspectral images band selection using multi-dictionary based sparse representation. Proceedings of the 2016 IEEE International Geoscience and Remote Sensing Symposium (IGARSS).

[19] Fang L., Li S., Kang X., Benediktsson J.A. (2014). Spectral–Spatial Hyperspectral Image Classification via Multiscale Adaptive Sparse Representation. IEEE Trans. Geosci. Remote Sens..

[20] Fang L., Wang C., Li S., Benediktsson J.A. (2017). Hyperspectral Image Classification via Multiple-Feature-Based Adaptive Sparse Representation. IEEE Trans. Instrum. Meas..

[21] Katiyar C., Yadav S.K., Mohammed Idris A. (2026). Hyperspectral Image Change Detection with Deep Learning: Methods, Trends, and Challenges. Remote Sens..

[22] Makantasis K., Karantzalos K., Doulamis A., Doulamis N. Deep supervised learning for hyperspectral data classification through convolutional neural networks. Proceedings of the 2015 IEEE International Geoscience and Remote Sensing Symposium (IGARSS).

[23] Zhong Z., Li Y., Ma L., Li J., Zheng W.S. (2022). Spectral-Spatial Transformer Network for Hyperspectral Image Classification: A Factorized Architecture Search Framework. IEEE Trans. Geosci. Remote Sens..

[24] Sun G., Pan Z., Zhang A., Jia X., Ren J., Fu H., Yan K. (2023). Large Kernel Spectral and Spatial Attention Networks for Hyperspectral Image Classification. IEEE Trans. Geosci. Remote Sens..

[25] Vaswani A., Shazeer N., Parmar N., Uszkoreit J., Jones L., Gomez A.N., Kaiser L., Polosukhin I. Attention is all you need. Proceedings of the 31st International Conference on Neural Information Processing Systems (NeurIPS).

[26] He K., Zhang X., Ren S., Sun J. Deep Residual Learning for Image Recognition. Proceedings of the 2016 IEEE/CVF Conference on Computer Vision and Pattern Recognition (CVPR).

[27] Huang G., Liu Z., Van Der Maaten L., Weinberger K.Q. Densely Connected Convolutional Networks. Proceedings of the 2017 IEEE/CVF Conference on Computer Vision and Pattern Recognition (CVPR).

[28] Dalal N., Triggs B. Histograms of oriented gradients for human detection. Proceedings of the 2005 IEEE Computer Society Conference on Computer Vision and Pattern Recognition (CVPR).

[29] Liu H., Bi W., Mughees N. (2025). Enhanced Hyperspectral Image Classification Technique Using PCA-2D-CNN Algorithm and Null Spectrum Hyperpixel Features. Sensors.

[30] Chen Q., Li Z., Yin J., Huang W., Zhan T. (2025). Local-Global Feature Extraction Network with Dynamic 3-D Convolution and Residual Attention Transformer for Hyperspectral Image Classification. IEEE J. Sel. Top. Appl. Earth Obs. Remote Sens..

[31] Li G., Ye M. (2025). 3D Wavelet Convolutions with Extended Receptive Fields for Hyperspectral Image Classification. arXiv.

[32] Roy S.K., Krishna G., Dubey S.R., Chaudhuri B.B. (2020). HybridSN: Exploring 3-D–2-D CNN Feature Hierarchy for Hyperspectral Image Classification. IEEE Geosci. Remote Sens. Lett..

[33] Chen S.Y., Hsu K.H., Kuo T.H. (2024). Hyperspectral Target Detection-Based 2-D–3-D Parallel Convolutional Neural Networks for Hyperspectral Image Classification. IEEE J. Sel. Top. Appl. Earth Obs. Remote Sens..

[34] Hong D., Han Z., Yao J., Gao L., Zhang B., Plaza A., Chanussot J. (2022). SpectralFormer: Rethinking Hyperspectral Image Classification with Transformers. IEEE Trans. Geosci. Remote Sens..

[35] Zou J., He W., Zhang H. (2024). PSFormer: Pyramid Superpixel Transformer for Hyperspectral Image Classification. IEEE Trans. Geosci. Remote Sens..

[36] Hu W., Shi W., Lan C., Li Y., He L. (2025). Hyperspectral Image Classification with Multi-Path 3D-CNN and Coordinated Hierarchical Attention. Remote Sens..

[37] Zhong Z., Liang C., Yang M., Wang D. (2026). LCTNet: Lightweight Convolution-Transformer Network for Hyperspectral Image Classification. IEEE J. Sel. Top. Appl. Earth Obs. Remote Sens..

[38] Sun J., Zhang H., Wang J., Sima H., Jin S. (2025). Hyperspectral Image Classification with Re-Attention Agent Transformer and Multiscale Partial Convolution. IEEE J. Sel. Top. Appl. Earth Obs. Remote Sens..

[39] Dong G., Basu A., Berretti S., Azari H. (2025). Medical Image Denoising via Explainable AI Feature Preserving Loss. Proceedings of the Smart Multimedia (ICSM 2024), Los Angeles, CA, USA, 28–30 March 2024.

[40] Banik B.C. (2025). Supervised-Blind Unmixing Comparison Technique with MiSiCNet and Deep Image Prior Model for Accurate Abundance Estimation in Hyperspectral Datasets. J. Future Artif. Intell. Technol..

[41] Chen G.Y., Krzyzak A., Xie W.F. (2022). Hyperspectral face recognition with histogram of oriented gradient features and collaborative representation-based classifier. Multimed. Tools Appl..

[42] Tang H., Li Y., Huang Z., Zhang L., Xie W. (2022). Fusion of Multidimensional CNN and Handcrafted Features for Small-Sample Hyperspectral Image Classification. Remote Sens..

[43] Zhang Z., Zhu X., Zhao D., Arun P.V., Zhou H., Qian K., Hu J. (2022). Hyperspectral Video Target Tracking Based on Deep Features with Spectral Matching Reduction and Adaptive Scale 3D Hog Features. Remote Sens..

[44] Baumgardner M.F., Biehl L.L., Landgrebe D.A. 220 Band AVIRIS Hyperspectral Image Data Set: June 12, 1992 Indian Pine Test Site 3. https://purr.purdue.edu/publications/1947/1.

[45] Zhong Y., Hu X., Luo C., Wang X., Zhao J., Zhang L. (2020). WHU-Hi: UAV-borne hyperspectral with high spatial resolution (H^2^) benchmark datasets and classifier for precise crop identification based on deep convolutional neural network with CRF. Remote Sens. Environ..

[46] Ma X., Kang X., Qin H., Wang W., Ren G., Wang J., Liu B. (2023). A Lightweight Hybrid Convolutional Neural Network for Hyperspectral Image Classification. IEEE Trans. Geosci. Remote Sens..

[47] Cui Y., Xia J., Wang Z., Gao S., Wang L. (2022). Lightweight Spectral–Spatial Attention Network for Hyperspectral Image Classification. IEEE Trans. Geosci. Remote Sens..

[48] Wang X., Fan Y. (2022). Multiscale Densely Connected Attention Network for Hyperspectral Image Classification. IEEE J. Sel. Top. Appl. Earth Obs. Remote Sens..

[49] Roy S.K., Jamali A., Chanussot J., Ghamisi P., Ghaderpour E., Shahabi H. (2025). SimPoolFormer: A two-stream vision transformer for hyperspectral image classification. Remote Sens. Appl..

[50] Cheng S., Chan R., Du A. (2024). CACFTNet: A Hybrid Cov-Attention and Cross-Layer Fusion Transformer Network for Hyperspectral Image Classification. IEEE Trans. Geosci. Remote Sens..

